# Prevalence and correlates of loneliness and social isolation in the oldest old: a systematic review, meta-analysis and meta-regression

**DOI:** 10.1007/s00127-023-02602-0

**Published:** 2023-12-15

**Authors:** André Hajek, Alina Volkmar, Hans-Helmut König

**Affiliations:** Department of Health Economics and Health Services Research, University Medical Center Hamburg-Eppendorf, Hamburg Center for Health Economics, Hamburg, Germany

**Keywords:** Aged, 80 and over, Loneliness, Prevalence, Social isolation, Social exclusion, Social disconnectedness

## Abstract

**Purpose:**

Conducting a systematic review, meta-analysis and meta-regression regarding the prevalence and correlates of loneliness and social isolation amongst the community-dwelling and institutionalised oldest old (80 years and over).

**Methods:**

Three electronic databases (PsycINFO, CINAHL and Medline) were searched, including studies from inception to January 5, 2023. An additional hand search was conducted by checking included studies’ references, and studies that cited included studies. We included observational studies describing the prevalence and (ideally) the correlates of loneliness, or social isolation, amongst individuals aged 80 years and over. Study design, operationalization of loneliness and social isolation, statistical analysis, characteristics of the sample and key findings were extracted. A random-effects meta-analysis was conducted.

**Results:**

We included 22 studies. The estimated prevalence of severe loneliness was 27.1% (95% CI: 23.7–30.4%). The estimated prevalence of moderate loneliness equalled 32.1% (95% CI: 15.8–48.4%). Moreover, the estimated prevalence of social isolation was 33.6% (95% CI: 28.9–38.2%). There was heterogeneity between the studies. Egger tests suggest the absence of potential publication bias. Meta-regressions showed that the heterogeneity could neither be attributed to the assessment of loneliness nor to the continent where the study was conducted.

**Conclusion:**

Loneliness and social isolation are important problems in the oldest old. In this age group, studies are required, in particular from regions outside Europe. Additionally, longitudinal studies are required to investigate the determinants of loneliness and social isolation amongst individuals aged 80 years and over. Studies using more sophisticated tools to quantify loneliness and social isolation are required.

**Supplementary Information:**

The online version contains supplementary material available at 10.1007/s00127-023-02602-0.

## Introduction

Loneliness (perceived discrepancy between current and desired social relationships [1]) and social isolation (lack of social activities [2]) are major threats to morbidity and longevity. For example, a recent meta-analysis [3] showed that both loneliness and social isolation were significantly associated with a greater risk of all-cause mortality: the pooled effect size for loneliness was 1.14 (95% CI: 1.08 to 1.20, *p* < 0.001) and the pooled effect size for social isolation was 1.32 (95% CI: 1.26 to 1.39, p < 0.001). Moreover, another meta-analysis [4] showed that poor social relationships were associated with a 32% (pooled relative risk: 1.32, 95% CI: 1.04 to 1.68) increase in stroke risk and a 29% (pooled relative risk: 1.29, 95% CI: 1.04 to 1.59) increase in coronary heart disease risk.

Both greater loneliness and greater social isolation are associated with a greater likelihood of having mental health disorders. For example, a former meta-analysis showed that loneliness had large effects on mental health outcomes (depression, anxiety, general mental health and suicidality) [5]. Large negative effects of social isolation on mental health (particularly amongst older people) have also been demonstrated [6].

A recent meta-analysis showed a pooled loneliness prevalence of 28.6% (95% CI: 22.9 to 35.0%) and a pooled social isolation prevalence of 31.2% (95% CI: 20.2 to 44.9%) amongst older adults aged 65 years and over (based on 15 countries of four continents: North America, South America, Asia and Europe) during the COVID-19 pandemic [7]. Higher pooled prevalence rates for loneliness were also identified amongst older adults (compared to young adults) in eastern European countries [8]. Moreover, two former meta-analyses showed higher loneliness levels in eastern and southern European countries, compared to northern European countries [8, 9].

Critical life events take place (e.g. loss of friends and relatives or health deteriorations) in later life, which can contribute to loneliness and social isolation amongst the oldest old (individuals aged 80 years and over) [10–12]. Moreover, social distancing during the pandemic can also have contributed to increased levels of loneliness and social isolation [13, 14]. To date, nine studies (e.g. [15, 16]) have examined the prevalence—and occasionally the correlates—of loneliness and social isolation amongst the oldest old. However, there has been no systematic review of studies (including meta-analysis and meta-regression) that systematically synthesises the present evidence. Therefore, our aim was to address this knowledge gap (by focussing on community-dwelling and institutionalised individuals aged 80 and over).

Specifically, the aim of this systematic review, meta-analysis and meta-regression was to identify the prevalence and correlates of loneliness and social isolation in the oldest old. Such knowledge is of great importance, particularly in view of the growing number of individuals in this age bracket. Additionally, our work may identify correlates of loneliness and social isolation. Furthermore, our work may clarify potential knowledge gaps and may thus inspire upcoming studies. Moreover, pooling of studies is possible by performing a meta-analysis. This can help deliver a more accurate overview compared to individual studies. A meta-regression can also assist in separating the influence of significant moderating factors (such as region in which the study was conducted, or tool used to quantify loneliness or social isolation).

## Methods

Our current work satisfied the Preferred Reporting Items for Systematic Reviews and Meta-Analysis (PRISMA) guidelines [17]. A PRISMA checklist can be found in Additional file 1. Furthermore, our work has been registered in the International Prospective Register of Systematic Reviews (PROSPERO, registration number: CRD42022339013). No amendments were made. In January 2023, an electronic search was conducted (three databases: Medline, CINAHL, PsycINFO). In Table 1, our search strategy for Medline is displayed (for the other databases: please see Additional file 2). The suitability was assessed by two reviewers (AV, AH) based on two steps (1: title/abstract screening and 2: full-text screening afterwards). Moreover, we conducted a hand search (i.e. (1) we examined the references of included studies and (2) we examined studies that cited the included studies). Grey literature was not searched. When perspectives on inclusion of studies differed, we used discussions to resolve this (if needed, a third party (HHK) was used). The same procedure applied to assessment of study quality and extracting data.

**Table 1:** Search strategy (Medline)

#1	Loneliness [MeSH Terms]
#2	Social isolation
#3	Social exclusion
#4	#1 OR #2 OR #3
#5	Oldest old
#6	Octogenarian
#7	Aged, 80 and over [MeSH Terms]
#8	#5 OR #6 OR #7
#9	#4 AND #8

Our inclusion criteria were:

- Cross-sectional and longitudinal observational studies identifying the prevalence of loneliness and social isolation amongst the oldest old (80 years and over), covering both, community-dwelling and institutionalized individuals
- Studies adequately assessing these constructs
- Studies published in peer-reviewed journals (German or English language)

It should be noted that the appropriate assessment of the constructs was strongly guided by the criteria of the COSMIN guidelines [18].

In contrast, studies exclusively focussing on samples with a certain disorder (e.g. individuals with Parkinson’s disease) were excluded. No restrictions were applied with respect to the time and place of the studies.

A pretest of 100 titles/abstracts was performed before the final inclusion criteria were determined. However, our inclusion criteria remained unchanged. Data were extracted by one reviewer (AV) and cross-checked by another (AH). Study design, definition and operationalization of loneliness, social isolation, characteristics of the sample, statistical analysis and key outcomes were extracted as data. If data were missing, study authors were contacted.

The quality of the studies was assessed using the established Joanna Briggs Institute (JBI) standardised critical appraisal instrument for prevalence studies [19]. The score ranges from 0 to 9 (whereby higher values reflect higher study quality and a lower general risk of bias). Study quality was independently assessed by two reviewers (AV and AH). A cut-off score for excluding studies from meta-analysis was not applied.

With respect to the meta-analysis, in order to pool proportions across the included studies, we used random-effects models because heterogeneity across studies was expected. Following given recommendations, heterogeneity between studies was estimated using the I^2^ statistic, with I^2^ values between 25 and 50% considered as low, 50% and 75% as moderate and 75% or more as high heterogeneity [20]. The well-known Stata tool ‘metaprop’ [21] was used to conduct meta-analysis.

It should be noted that loneliness was grouped into “not lonely”, “moderately lonely” and “severely lonely” largely following the procedure proposed by Gardiner et al. [22]. For further details, please see the Additional file 3.

With regard to social isolation, the few single studies dealing with social isolation usually simply distinguished between the presence of social isolation and the absence of it. Therefore, we maintained this dichotomy for the meta-analysis. The detailed presentation of the dichotomization of social isolation in the single studies is provided in Table 2.

**Table 2:** Study overview and key findings

Author (Year)	Country	Assessment of loneliness and/or social isolation	Study type	Sample characteristics and target population; time of data collection	Sample size and age in total sample	Results: *n* = prevalence of loneliness and/or social isolation (%)	Results: prevalence stratified by sex	Results: correlates and associated factors
Amaral et al. (2021)	Portugal	Semi-structured Interviews; Loneliness was assessed through the question: “Do you feel alone?”, taken from “UCLA Loneliness Scale”	Cross-sectional	Oporto Centenarian Study (PT100) and Beira Interior Centenarian Study (PT100 BI), centenarians from Covilhã and Oporto Time of data collection: December 2012 to May 2014	*n* = 48 centenarians (18 centenarians from the PT100 Oporto Centenarian Study and 30 centenarians from the PT100 Beira Interior Centenarian Study), mean age: 100.8 years, SD = 1.2 years, proportion of female participants: 83.3%	*n* = 11 (22.9%) were severely lonely; *n* = 14 (29.2%) were moderately lonely, *n* = 23 (48%) were not lonely (rarely and never)	Not reported	Not reported
Brittain (2017)	United Kingdom	loneliness was assessed by “single-question self-rating” scale by Victor, Scambler and Bond^1^, response options: “always”, “often”, “sometimes”, “never”	Longitudinal	Newcastle 85 + dataset, longitudinal study with participants over 85 years Time of data collection: Baseline: June 2006 to October 2007 (first wave: 18 months later; second wave: 36 months after baseline)	*n* = 750, Age: 85 years (all participants were born in 1921), SD: not reported, proportion of female participants: *n* = 460 (61.3%)	9.73% were severely lonely; 33.6% were moderately lonely, 56.67% were not lonely	Women 12.39% were severely lonely; 39.13% were moderately lonely, 48.48% were not lonely Men 5.52% were severely lonely, 24.83% were moderately lonely, 69.66% were not lonely	Significant correlates of higher loneliness (amongst other things, based on repeated measures ordinal logistic regression): • Living alone (OR: 4.43, 95% CI: 3.50–5.36) and being institutionalised (OR: 3.1, 95% CI: 1.91–4.29) compared to not living alone in the community • 5 + years of widowhood (OR: 0.44, 95% CI: 0.26–0.73) compared to not being widowed • Severe depression (OR: 7.43, 95% CI: 5.92–8.95) compared to the absence of depression Disability score of 1–6 (OR: 1.47, 95% CI: 1.24–1.79) compared to disability score of zero
Chou (2005)	China	Interviews; loneliness was assessed by the question: “How often do you feel lonely?”, responses were given on a five-point scale ranging from 0 = never to 4 = always (0 = never, seldom or sometimes feel loneliness and 1 = frequently or always feel lonely)	Cross-sectional	Sample of 1903 Chinese elderly people aged 60 years or above living in Hong Kong Time of data collection: 1996	*n* = 183, age: 80 and above (oldest-old), mean age: not reported, SD: not reported, proportion of female participants: 57.9%	Overall 13.1% were lonely Non-depressed 3.2% were lonely Depressed 35.1% were lonely	Not reported	Multiple logistic regression: Presence of loneliness (independent variable) was significantly associated with a higher likelihood of depression (OR: 18.39, 95% CI: 4.08–82.93)
Costenoble (2022)	Belgium	Questionnaire; Loneliness was assessed by 2 questions out of the 6-item scale by de Jong Gierveld and Van Tilburg, Questions: “Do you experience emptiness?”, and “There are plenty of people with whom I feel closely connected to”, response options: yes (1), more or less (2), no (3)	Cross-sectional	Participants of BrUssels sTudy on The Early pRedictors of FraiLtY (BUTTERFLY); community-dwelling octogenarians Time of data collection: During the first COVID-19 lockdown (not further specified)	*n* = 215, mean age: 86.5 years, SD: 3.0, proportion of female participants: *n* = 98 (46%)	Experiencing emptiness Yes: 10% More or less: 22% No: 68% Being closely connected to Yes: 72% More or less: 22% No: 6%	Not reported	Multiple linear regression: Not experiencing emptiness compared to experiencing emptiness (independent variable) was associated with higher quality of life (β = .65, 95% CI: .32-.98)
Dahlberg (2018)	Sweden	Structured interviews, Loneliness was assessed through question: “Are you ever bothered by feelings of loneliness?”, response options: “nearly always”, “often”, “seldom”, “almost never”; response options “nearly always” and “often” were dichotomised into “frequently lonely” and response options “seldom” and “almost never” were dichotomised into “rarely lonely”	Longitudinal	Swedish Level of Living Survey (LNU), Swedish Panel Study of Living Conditions of the Oldest Old (SWEOLD) Time of data collection: 2002 and 2011 (SWEOLD); 1981 and 1991 (LNU)	*n* = 823, mean age: 82.4 years, SD: not reported, proportion of female participants: 59%	12.8% were lonely	Not reported	Not reported
Eliasen (2022)	Faroe Islands (Denmark)	Telephone interview; Questions to assess loneliness: “Do you feel lonely?” and “Would you like more social contact?”, response options: “yes” or “no”	Cross-sectional	Faroese Septuagenarians cohort (1131 citizens born between January 1934 and August 1937, aged 70–74 years); population-based study Time of data collection: December 2017 to January 2019 and June to July 2020	*n* = 220 mean age: 84.4 years, SD: 1.0 years, proportion of female participants: *n* = 118	Before COVID-19 6.8% were lonely, 93.2% were not lonely; During COVID-19 21.8% were lonely, 78.2% were not lonely	Not reported	Not reported
Ha (2019)	United States	Interviews; social isolation was measured using a modified version of 18-item Lubben Social Network Scale (9 items were used), 3 questions each from 3 different networks (relatives, friends, neighbours); questions: “How many [relatives/friends/neighbours] (a) do you see or hear from at least once a month, (b) do you feel close to such that you could call on them for help, and (c) do you feel at ease with that you can talk about private matters?”, response categories: 0 = none, 1 = one, 2 = two, 3 = three or four, 4 = five through eight, and 5 = nine or more; 1. Scores were summed 2. dichotomous variable, score of 18 or less was considered as at risk of social isolation (range 0–45) scores of 12 or less on the LSNS-6 and scores of 18 or less on the LSNS-9 scale 3. sub-scales for each network (each sub-scale ranged from 0 to 15)	Cross-sectional	Hospitalist Study (HS); participants aged 60 years and over who were admitted to inpatient units of hospital in Chicago Time of data collection: July 2011 to June 2013	Proportion of female participants: not reported 80–89 years *n* = 567, SD: not reported 90–108 years *n* = 187, SD: not reported	80–89 years (n = 567) 34.1% were socially isolated on LNSN-6 scale; 21.4% were isolated on LSNS-9 < 12 scale, 47.8% were isolated on LSNS-9 < 18 scale 90–108 years (n = 187) 43.8% were socially isolated LNSN-6 scale, 23.6% were isolated on LSNS-9 < 12 scale, 55.8% were isolated on LSNS-9 < 18 scale	Not reported	Not reported
Hajek (2021)	Germany	Social isolation was quantified using the 6-item version of the Lubben Social Network Scale, absence of social isolation if LSNS-6 ≥ 12; presence of social isolation if LSNS-6 < 12	Longitudinal	AgeQualiDe study, oldest-old primary care patients (85 +) Time of data collection: Year 2014/2015 to year 2016/2017	FU wave 7 *n* = 640, mean age: 88.8 years, SD: 2.9 years, proportion of female participants: *n* = 434 (67.8%), FU wave 8 *n* = 627, mean age: 89.6 years, SD: 2.8 years, proportion of female participants: *n* = 426 (67.9%) FU wave 9 *n* = 525, mean age: 90.4 years, SD: 2.7 years, proportion of female participants: *n* = 362 (68.9%)	FU wave 7 30.8% were socially isolated FU wave 8 35.9% were socially isolated FU wave 9 36.8% were socially isolated	Not reported	Conditional logistic fixed effects regression: Over time, the occurrence of social isolation (independent variables) was found to be associated with an increased likelihood of experiencing self-care problems (OR: 1.92, 95% CI: 1.01–3.65), pain/discomfort (OR: 2.01, 95% CI: 1.16–3.48) at an individual level
Holmen (1994)	Sweden	Interviews; Experienced loneliness was investigated with the question: "Do you experience loneliness?", response options: “Yes”, “No”, “Often”, “Sometimes”, “Seldom”, “Never”, “Do not know”, response options were dichotomized into “often + sometimes” and “seldom + never”, people who responded “Do not know” were excluded	Longitudinal	Kungsholmen longitudinal study, 2368 participants aged 75 years and over living in Stockholm, data in study comprises 211 persons aged 90 years and over; Time of data collection: Starting in 1987 (follow-up took place 2.5 years later)	*n* = 185, age: 90 years and over, mean age: not reported, SD: not reported, proportion of female participants: not reported	52.4% were lonely	Not reported	Not reported
Jacobs (2014)	Israel	Questionnaire; Loneliness was assessed with the question: “How often do you feel lonely? “, response options: “never”, “rarely”, “often”, “very often”, dichotomized into “not lonely” and „lonely “	Cohort study	Jerusalem Longitudinal Study, birth cohort of Jerusalem residents (born between June 1920 and May 1921) Time of data collection: 1990 (baseline) to 2010/2011 (phase 4)	Age 85 (2005) *n* = 1224, SD: not reported, proportion of female participants: *n* = 670 Age 90 (2010) *n* = 673, SD: not reported, proportion of female participants: *n* = 387	Not reported as total number, directly stratified into men and women	Age 85 (2005) Women: 53.4% Men: 32.2% Age 90 (2010) Women: 53.2% Men: 35.2%	Not reported
Kim (2022)	United States	Interviews; Loneliness and social isolation was measured using a 3-item version of UCLA loneliness scale, Questions: “How often do you feel you lack companionship?”, “How often do you feel left out?”, “How often do you feel isolated from others?”, response options 1 = hardly ever, 2 = some of the time, 3 = often, scores were summed (range 3–9), loneliness was categorised as a binary variable, with a value of 0 assigned when the sum falls between 3 and 5 and a value of 1 assigned when the sum falls between 6 and 9	Longitudinal	Longitudinal cohort study (HEPESE), Mexican American participants aged 65 years and older living in Texas, New Mexico, Colorado, Arizona and California Time of data collection: 2010 to 2011 (wave 7), 2012 to 2013 (wave 8), and 2016 (wave 9)	Wave 7 (2010–2011) *n* = 1126, mean age: 85.9 years, SD: 4.0 years, proportion of female participants: 65%	Wave 7 25% were lonely	Not reported	Not reported
Kotian (2018)	India	Social isolation was considered as present if participants responded “never” to all 4 of the activities within the last 12 month: (1) attending a public meeting, (2) attending any group/club/organisational meeting, (3) attending any religious programme, (4) visiting friends or relatives	Cross-sectional	Building Knowledge Base on Population Ageing in India (BKPAI) survey Time of data collection: 2011	80–89 years *n* = 838, mean age: not reported, SD: not reported, proportion of female participants: not reported ≥ 90 years*n* = 166 mean age: not reported, SD: not reported, proportion of female participants: not reported	80–89 years 33.7% were socially isolated, 66.3% were not socially isolated ≥ 90 years50% were socially isolated, 50% were not socially isolated	Not reported	Not reported
Lay-Yee (2021)	New Zealand	Questionnaire; Loneliness was assessed by the question: “Would you say that you—always, often, sometimes or never feel lonely?”, response options: “never”, “sometimes”, “often”, “always”, response options were dichotomized into: “lonely” (always, often, sometimes) and “not lonely” (never)	Longitudinal	LiLACS NZ (“Life and Living in Advanced Age: a Cohort Study in New Zealand”), participants with advanced age, non-Māori aged 85 years and Māori aged 80–90 years Time of data collection: 2010 (wave 1) to 2015 (wave 6)	Māori *n* = 254, mean age: not reported, SD: not reported, proportion of female participants: *n* = 154 (60.6%) Non-Māori *n* = 398, mean age: not reported, SD: not reported, proportion of female participants: *n* = 210 (52.8%)	Māori 39.8% were lonely, 60.2% were not lonely Non-Māori 28.1% were lonely, 71.9% were not lonely	Māori Men 38.0% were lonely, 62.0% were not lonely Women 40.9% were lonely, 59.1% were not lonely Non-Māori Men 25.0% were lonely, 75.0% were not lonely Women31.0% were lonely, 69.0% were not lonely	Mixed-effects models: - Amongst other things, being lonely compared to not being lonely (independent variable) was associated with a lower likelihood of having a high life satisfaction (amongst Māori, OR: 0.35, 95% CI: 0.21 to 0.59; amongst non-Māori, OR: 0.39, 95% CI: 0.26 to 0.58)
Leitch (2018)	New Zealand	Electronic assessment tool with 236-item, Loneliness was assessed by the response to the statement: “Says or indicates that he/she feels lonely”, response options: “Yes”, “No”	Cross-sectional	Sample of New Zealanders, aged 65 years and older who live independently in the community and who have completed the international Resident Assessment Instrument–Home Care (interRAI-HC) Time of data collection: January 2013 to November 2017	Age: 80–84 *n* = 17,284, mean age: not reported, SD: not reported, proportion of female participants: not reported Age: 85–89 *n* = 16,850, mean age: not reported, SD: not reported, proportion of female participants: not reported Age: 90–94 *n* = 8,804, mean age: not reported, SD: not reported, proportion of female participants: not reported Age: 95–99*n* = 1923, mean age: not reported, SD: not reported, proportion of female participants: not reported age: 100–109 *n* = 191, mean age: 100.9 years, SD: 1.2 years, proportion of female participants: *n* = 136 (71.2%)	80–84 years 19.4% were lonely 85–89 years 19.9% were lonely 90–94 years 20.5% were lonely 95–99 years 17.4% were lonely 100–109 years 14.7% were lonely	Not reported	Poisson regression: Significant correlates of higher loneliness: - Younger age (81.4 years compared to 100.9 years, RR: 0.68, 95% CI: 0.58 to 0.79) - Being Asian compared to being European (RR: 1.29, 95% CI: 1.20 to 1.40) - Certain marital statuses, e.g. being widowed compared to being married (RR: 1.06, 95% CI: 1.00 to 1.12) - Absence of family support compared to the presence of family support (RR: 0.83, 95% CI: 0.79 to 0.86) - Living alone, e.g. compared to living with partner (RR: 0.37, 95% CI: 0.35 to 0.40) - Presence of, e.g. moderate/severe depression compared to the absence of depression (RR: 3.45, 95% CI: 3.22 to 3.70)
Liu (2012)	China	Interviews; Loneliness was assessed by the question: “Do you often feel lonely and isolated?”, response options range from 1 (always or very good) to 5 (never or bad), to dichotomize loneliness, responses indicating feeling lonely and isolated (always/often) are coded as 1, all other responses are coded as 0	Cross-sectional	Chinese Longitudinal Healthy Longevity Survey (CLHLS) Time of data collection: 1998 (wave 1), 2000 (wave 2), and 2002 (wave 3)	20,156 Respondents aged 80 and older Community residing *n* = 19,047, mean age: 92.3 years, SD: 7.7 years, proportion of male participants: *n* = 7405 (38.9%) institutionalised *n* = 1,109, mean age: 89.4 years, SD: 7.4 years, proportion of male participants: *n* = 495 (44.6%)	Community residing 15.9% were lonely institutionalised13.6% were lonely	Not reported	Not reported
Nobrega (2022)	Brazil	Interviews; questionnaire of Health, well-being and Ageing (*Saúde, Bem-Estar e Envelhecimento* (SABE), response options to question regarding loneliness (yes/no), social isolation (yes/no)	Cross-sectional	Participants living in Brejo dos Santos/PB and São Paulo/SP Time of data collection: May 2017 (in Brejo dos Santos) and March to June 2016 (in São Paulo)	*n* = 417 oldest-old persons, age: 80- 102 years SABE-PB *n* = 179, age: 80–102 years residing in Brejo dos Santos/PB, mean age: 85.5 years, SD: 5 years, proportion of female participants: *n* = 98 (54.7%) SABE-SP *n* = 238, age: 80–101 years in Sao Paulo, mean age: 86.8 years, SD: 4.73 years, proportion of female participants: *n* = 168 (70.6%)	SABE-PB 54.9% were lonely, 45.1% were not lonely; 35.7% were socially isolated, 64.3% were not socially isolated SABE-SP 41.2% were lonely, 58.8% were not lonely;24.0% were socially isolated, 76.0% were not socially isolated	Not reported	Not reported
Nyqvist (2013)	Finland, Sweden	Structured interviews; loneliness was assessed through the question: “Do you ever feel lonely?”, response options: “often”, “sometimes”, “seldom”, “never”, response options “seldom” and “never” were dichotomized into “not lonely” and “often” and “sometimes” into “lonely”	Cross-sectional	Gerontological Regional Database and Resource Centre project (GERDA) which is a continuation of Umeå 85 + study, participants were older people in Bothnia region (on both sides of Gulf of Bothnia, in Sweden (Västerbotten) and Finland (Pohjanmaa)) Time of data collection: 2005 to 2007	*n* = 48, Age: 85 years and over, mean age: not reported, SD: not reported, proportion of female participants: *n* = 334 (69.2%) At home *n* = 334 institution *n* = 149	At home 8.1% were severely lonely, 60.5% were moderately lonely (sometimes and seldom together), 31.4% were not lonely Institution 18.8% were severely lonely 57% were moderately lonely (sometimes and seldom), 24.2% were not lonely	Not reported	Multiple logistic regression: Significant correlates of higher loneliness amongst community-dwelling individuals: - living alone compared to living with someone (OR: 7.80, 95% CI: 3.77 to 16.15) - presence of probable depression compared to the absence (OR: 4.90, 95% CI: 2.40 to 10.02) Significant correlates of higher loneliness amongst institutionalised individuals: - presence of probable depression compared to the absence (OR: 9.43, 95% CI: 3.44 to 25.85)
Stek (2005)	The Netherlands	Interviews; loneliness was assessed by De Jong Gierveld loneliness tool (11-item questionnaire), 3 points and more were considered as perceived loneliness	Prospective population-based study	Leiden 85-Plus Study (prospective population-based Study), participants were members of 1912 to 1914 birth cohort living in Leiden (enrolled in month of their 85th birthday) Time of data collection: 1997 to 2002	*n* = 476 Age: 85 years, mean age: 85, SD: 0, proportion of female participants: *n* = 305 (64.1%) Depressed *n* = 109 Not depressed *n* = 367	Perceived loneliness was present in 25% of the participants Depressed Amongst the depressed participants, 52% were lonely, amongst the depressed participants, 48% were not lonely Not depressed Amongst the not depressed participants, 17% were lonely, amongst the not depressed participants, 83% were not lonely	Not reported	Not reported
Tigani (2012)	Greece	Face-to-face interviews, Question: “Do you feel lonely or abandoned?”, response options “yes” or “no”	Cross-sectional	Greek centenarians living in 10 out of 11 geographic divisions of Greece Time of data collection: 2007 to 2010	*n* = 400, age: 100–109 years old, mean age: 101.9 years, SD: not reported, proportion of female participants: *n* = 251 (62.8%)	54.7% were lonely, 45.3% were not lonely	Not reported	Multiple logistic regression: - The presence of loneliness (compared to the absence; independent variable) was associated with a higher likelihood of poor self-rated health (OR: 2.23, 95% CI: 1.24 to 4.01)
Timmermans (2019)	The Netherlands	Interviews; Loneliness was assessed with De Jong-Gierveld Loneliness Scale (range: 0–11), score of 3 or higher was considered indicative of loneliness; Social isolation was defined as the absence of daily contact with anyone from the participants' personal social network, participants who lived with a partner or someone else were considered not socially isolated	Cohort study	Longitudinal Ageing Study Amsterdam (LASA), participants from 11 municipalities across 3 regions in the Netherlands Time of data collection: 1992 to 1993 (baseline), up to 2011/2012 (wave 7)	*n* = 603, age: 85–94 years, mean age: 88.9 years, SD: not reported, average proportion of female participants: 63.7% 2001/02 *n* = 308, mean age: 88.8 years, SD: 2.6, proportion of female participants: 62% 2005/06 *n* = 226, mean age: 89 years, SD: 2.7, proportion of female participants: 62.8% 2008/09 *n* = 198, mean age: 89.2 years, SD: 2.9, proportion of female participants: 65.2% 2011/12 *n* = 199, mean age: 88.7 years, SD: 2.7, proportion of female participants: 65.8%	**Loneliness** 2001/02 53.6% were lonely 2005/06 48.5% were lonely 2008/09 47.7% were lonely 2011/12 50% were lonely **Social isolation** 2001/0233.0% were socially isolated 31.9% were socially isolated, 2008/0925.5% were socially isolated, 2011/1226.7% were socially isolated2005/06	Not reported	Not reported
Wang (2019)	United Kingdom	Interviews; single-item scale, Loneliness was assessed using the Question: “Do you feel lonely?”, response options: “not at all lonely”, “slightly lonely”, “lonely”, “very lonely”, response options “lonely” and “very lonely” were dichotomized into “lonely”	Longitudinal	Cambridge City over-75 s Cohort Study (CC75C), participants 75 years and older, data was taken from wave 3–5 Time of data collection: Wave 3 to wave 5 (exact years not further specified)	*n* = 665, age: 80 years and older, mean age: not reported, SD: not reported, proportion of female participants: *n* = 458 (69%)	59% were not lonely, 16% were moderately lonely, 25% were severely lonely	Men 71% were not lonely, 12% were moderately lonely, 17% were severely lonely Women 53% were not lonely, 18% were moderately lonely, 29% were severely lonely	Negative binomial regression: • Feeling slightly lonely (compared to not lonely; independent variable) was positively associated with GP visits (IRR: -0.5, 95% CI: -0.8 to -0.2) - Feeling lonely (time-varying; compared to not lonely); independent variable was associated with a higher likelihood of community nurse (IRR: 3.4, 95% CI: 1.4 to 8.7) and meals on wheels (IRR: 2.5, 95% CI: 1.1 to 5.6)
Zaccaria (2022)	United States	Interviews; Social isolation was assessed using the 6-item Lubben Social Network Scale (LSNS-6), response options: 0 (none) to 5 (nine or more), (range: 0–30), score more than 12 was considered as social isolation Loneliness was assessed using a 5-item version of UCLA Loneliness Scale, response options ranged from 1 = never, 2 = rarely, 3 = sometimes to 4 = often	Cross-sectional	Subgroup of Fordham Centenarian Study Time of data collection: not clarified	*n* = 94, age: 95–107 years, mean age: 99.6 years, SD: 2.4, proportion of female participants: *n* = 73 (77.7%)	29.8% were lonely and isolated, 21.3% were isolated but not lonely, 20.2% were lonely but not isolated, 28.7% were neither lonely nor isolated	Not reported	Not reported

Regarding meta-regression, we used the ‘meta regress’ command. More precisely, we performed a random-effects meta-regression with restricted maximum likelihood. Knapp–Hartung adjustment was applied for the standard errors [23]. The coefficients were recalculated. The reason is that the coefficients in the meta-regressions were initially scaled as double arcsine values (rather than proportions) (following Lipsey and Wilson [24]). Meta-regressions were conducted to identify the heterogeneity sources [25].

To detect a potential publication bias, a funnel plot as well as the Egger test (p < 0.05 indicates publication bias) were conducted [26]. Stata 16.1 (College Station, TX, USA) was used in our current study.

## Results

### Study overview

**Fig. 1:**
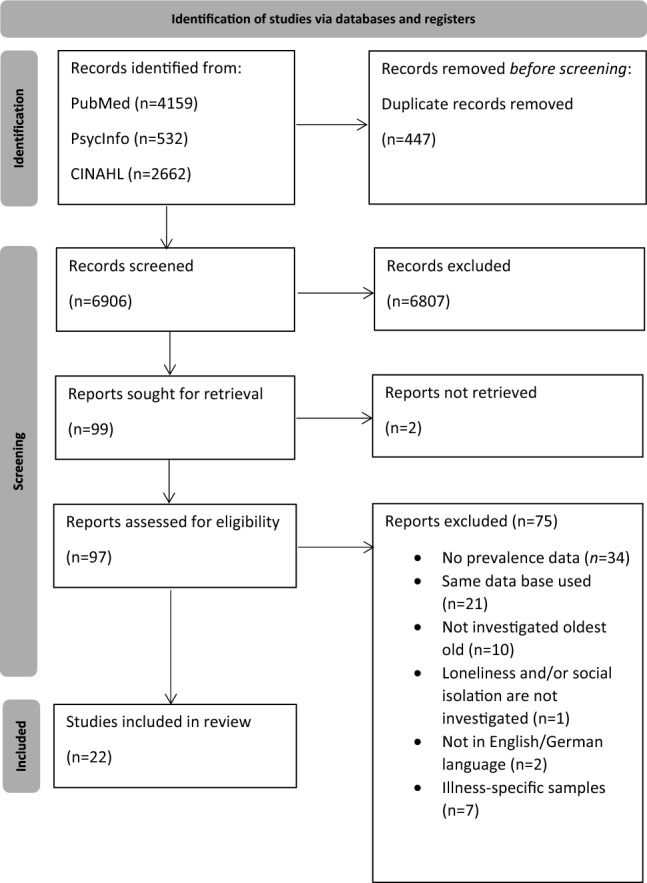
Flow chart

A flow chart is given in Fig. 1 [17]. More precisely, this figure illustrates the flow of information across the various stages of our systematic review and meta-analysis.

After eliminating duplicates, a total of 6,906 studies underwent screening, specifically through the evaluation of titles and abstracts. During this initial phase, the most prevalent reason for exclusion was the absence of reported data on loneliness or social isolation prevalence amongst the oldest individuals. In the subsequent step, which involved assessing the full text of selected studies, some distinct reasons for exclusion were identified (e.g. not reporting prevalence data or not examining oldest old individuals). When different studies used the same dataset, we selected the study that used the most comprehensive dataset (see also: [27, 28]). Ultimately, our present systematic review incorporated a total of 22 studies [15, 16, 29–48], with all of these studies included in the meta-analysis. The hand search did not reveal additional studies. Table 2 describes important characteristics of the studies and key findings. Within the scope of our analysis, three studies investigated the factors associated with loneliness [16, 40, 43], and their adjusted results are presented in Table 2.

Data were from Europe (*n* = 12, two studies each from the Netherlands, Sweden, the United Kingdom, one study each from Belgium, Faroe Islands, Finland/Sweden, Germany, Greece, Portugal), South America (*n* = 1 from Brazil), Asia (*n* = 4, two studies from China, one study from Israel and one study from India), North America (*n* = 3, all from the United States) and Oceania (*n* = 2, both from New Zealand). In sum, 13 studies had a cross-sectional design, and nine studies had a longitudinal design. Large, representative surveys were used in 17 studies. Given the fact that data were collected amongst the oldest old, it has to be noted that twelve had a large sample size (with sample sizes in the higher three- or four-digit range, e.g. 600 or higher). The proportion of women ranged from about 60% to 80% in 15 studies and the average age, if reported, ranged from 80 to 90 years in nine studies. Overall, 15 studies used single item measures to quantify loneliness. The remaining four studies used different versions of the UCLA-tool and the De Jong Gierveld tool (11-item version). Three studies examining social isolation used the LSNS-6, whereas the remaining three studies used single-item measures and a self-developed tool (based on four activities). Additional file 4 displayed the frequency for the tools used to quantify loneliness and social isolation, respectively.

The studies were published between 1994 and 2022 and 14 out of the 22 studies were published in or after 2018 (3 times: 2018, 3 times: 2019, 3 times: 2021 and 5 times: 2022). Data collection took place during the first COVID-19 lockdown in one study [30]. In a second study, data collection took partly place during the COVID-19 pandemic (i.e. June to July 2020) [32]. Further details are shown in Table 2.

### Correlates of loneliness

Three studies examined the correlates of loneliness [16, 40, 43]. All three studies found that living alone and the presence of depression are associated with a greater likelihood of loneliness [16, 40, 43]. Two (out of two) studies found that being widowed is also associated with a greater likelihood of loneliness [16, 40].

It may be worth noting that some other studies used loneliness as independent variable. They found that greater loneliness is associated with a greater likelihood of depression [29], lower quality of life [30], lower life satisfaction [39], and poor self-rated health [45].

### Correlates of social isolation

The correlates of social isolation were not examined by any of the studies. In contrast, one study used social isolation as independent variable and found that the occurrence of social isolation was associated with an increased likelihood of experiencing self-care problems (OR: 1.92, 95% CI: 1.01–3.65), and pain/discomfort (OR: 2.01, 95% CI: 1.16–3.48) over time [34].

### Meta-analysis and meta-regression

**Fig. 2:**
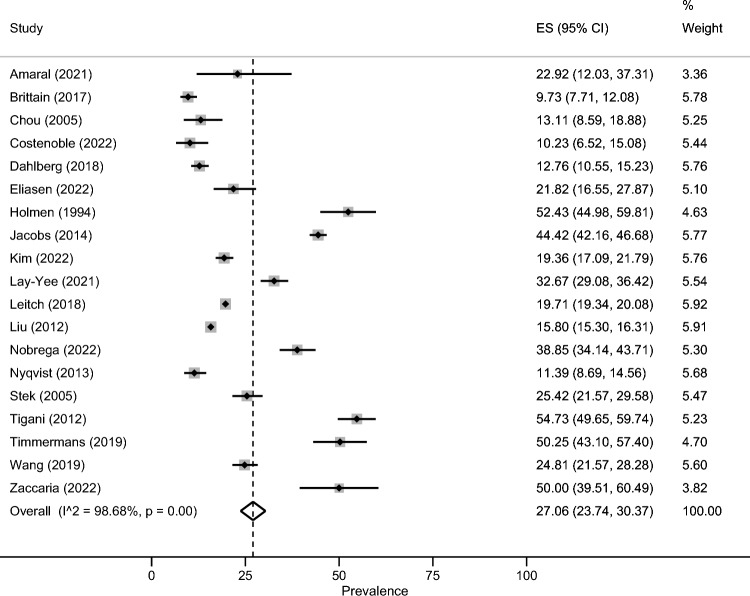
Meta-analysis (severe loneliness)

**Fig. 3:**
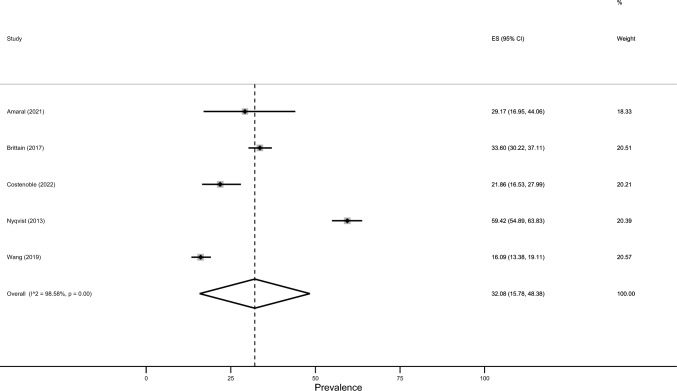
Meta-analysis (moderate loneliness)

The estimated prevalence of severe loneliness was 27.1% (95% CI: 23.7–30.4%; Fig. 2). There was significant heterogeneity between studies (*I*^2^ = 98.7%, p < 0.001). The estimated prevalence of moderate loneliness equalled 32.1% (95% CI: 15.8–48.4%, Fig. 3; *I*^2^ = 98.6%, p < 0.001). Moreover, the estimated prevalence of social isolation was 33.6% (95% CI: 28.9–38.2%, *p* < 0.001; *I*^2^ = 88.7%, *p* < 0.001).

With regard to sex-stratified prevalences for loneliness: The estimated prevalence of severe loneliness was 33.6% amongst women (95% CI: 6.6–60.7%, *I*^2^ = 99.4, *p* < 0.001), whereas it was 22.7% amongst men (95% CI: 3.0%–42.4%, *I*^2^ = 99.0%, *p* < 0.001; see Additional file 5 for meta-analysis stratified by sex).

Furthermore, our meta-regression analysis indicated that the assessment tool for loneliness and the continent in which the study took place did not significantly influence the prevalence of loneliness (Table 3; a model that considers each category of the two variables individually can be found in Additional file 6). Please note: The coefficients of the regression reflect the predicted change in the logit given a 1-unit change in the moderator variable.

**Table 3:** Meta-regression analysis of factors affecting heterogeneity (severe loneliness)

Variables	Coefficient (95% confidence interval)	*p* value
Assessment:—De Jong-Gierveld/UCLA (Reference category: Single-item)	.55 (− .43–1.54)	.26
Continent: South America/North America/Asia/Oceania (Reference category: Europe)	.15 (− .67–.96)	.71

In a robustness check, we also added the risk of bias scores in a meta-regression (to compare findings from studies at lower and higher risk of bias) [49]. However, this factor also did not achieve statistical significance (*p* = 0.18). We refrained from doing a meta-regression analysis with prevalence of social isolation due to the small number of studies included. We therefore conducted meta-analysis with prevalence of social isolation for subgroups (by region; by tool used to quantify social isolation; by risk of bias). These findings can be found in Table 4.

**Table 4:** Subgroup analysis of the pooled prevalence of social isolation

Characteristics	Subgroups	Number of studies	Prevalence	95% CI	I^2^ (%), *p* value
Region	North America	2	33.5	31.6 to 35.3	0.00, *p* = .
	Europe	2	33.6	30.2 to 37.0	0.00, *p* = .
	Other regions (Asia, South America)	2	32.0	29.6 to 34.4	0.00, *p* = .
Instrument used to quantify social isolation	LSNS-6	3	38.3	31.5 to 45.0	85.8,* p* < .01
	Other tools (2x: Single item, 1x: based on four activities)	3	29.1	20.2 to 37.9	92.4, *p* < .01
Quality assessment score	Score of 7 or 8	4	36.6	30.5 to 42.8	83.0, *p* < .01
	Score of 5 or 6	2	31.3	29.6 to 33.0	0.00, *p* = .

**Fig. 4:**
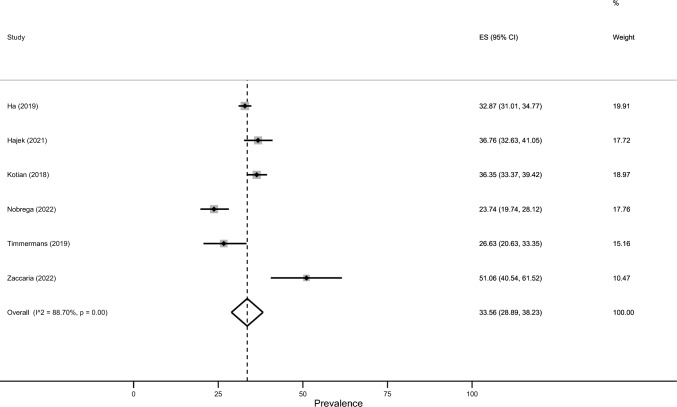
Meta-analysis (social isolation)

**Fig. 5:**
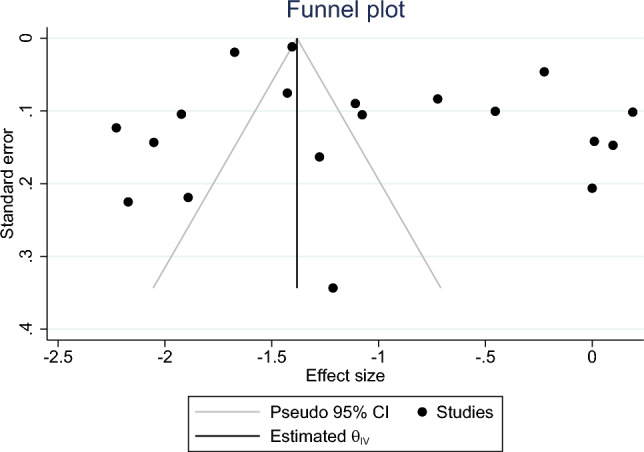
Funnel plot

The funnel plot (Fig. 4) suggested a potential asymmetry (for loneliness). However, the Egger test (*p* = 0.75) suggested no potential data asymmetry; this indicates the absence of potential publication bias (Fig. 5).

### Quality assessment/risk of bias assessment

Table 5 shows the quality assessment/risk of bias evaluation. The scores varied from 2 to 8 (mean score: 6.4, SD: 1.6), indicating a moderate to good level, and a comparably low danger of bias. The most common limitation was that the response rate was not clearly displayed/unclear handling of low response rate (all studies).

**Table 5:** Quality assessment/risk of bias assessment

Study	1	2	3	4	5	6	7	8	9	Total (from 1 to 9; higher scores indicate less risk of bias)
Amaral (2021)	Y	N	N	Y	Y	Y	N	N	U	4
Brittain (2017)	Y	Y	Y	Y	Y	Y	Y	Y	U	8
Chou (2005)	Y	Y	Y	N	Y	Y	Y	N	U	6
Costenoble (2022)	Y	Y	Y	Y	Y	Y	U	Y	U	7
Dahlberg (2018)	Y	Y	Y	N	Y	Y	U	Y	U	6
Eliasen (2022)	Y	U	Y	Y	U	Y	U	Y	U	5
Ha (2019)	N	Y	Y	Y	U	Y	U	Y	U	5
Hajek (2021)	Y	Y	Y	Y	Y	Y	U	Y	U	7
Holmen (1994)	U	U	Y	N	U	Y	N	U	U	2
Jacobs (2014)	Y	Y	Y	Y	Y	Y	Y	Y	U	8
Kim (2022)	Y	U	Y	Y	Y	Y	U	Y	U	6
Kotian (2018)	Y	Y	Y	Y	Y	Y	Y	Y	U	8
Lay-Yee (2021)	Y	Y	Y	Y	U	Y	U	Y	U	6
Leitch (2018)	N	U	Y	Y	U	Y	U	Y	U	4
Liu (2012)	Y	Y	Y	Y	Y	Y	Y	Y	U	8
Nobrega (2022)	Y	Y	Y	Y	U	Y	U	Y	U	6
Nyqvist (2013)	Y	Y	Y	Y	Y	Y	Y	Y	U	8
Stek (2005)	Y	Y	Y	Y	Y	Y	Y	Y	U	8
Tigani (2012)	Y	N	Y	Y	Y	Y	U	Y	U	6
Timmermans (2019)	Y	Y	Y	Y	N	Y	Y	Y	U	7
Wang (2019)	Y	Y	Y	Y	Y	Y	Y	Y	U	8
Zaccaria (2022)	Y	Y	Y	Y	Y	Y	U	Y	U	7

The Joanna Briggs Institute (JBI) standardised critical appraisal instrument for prevalence studies was used. Y: Yes, N: No, U: Unclear; 1: Was the sample frame appropriate to address the target population? 2: Were study participants sampled in an appropriate way? 3: Was the sample size adequate? 4: Were the study subjects and the setting described in detail? 5: Was the data analysis conducted with sufficient coverage of the identified sample? 6: Were valid methods used for the identification of the condition? 7: Was the condition measured in a standard, reliable way for all participants? 8: Was there appropriate statistical analysis? 9: Was the response rate adequate, and if not, was the low response rate managed appropriately?

## Discussion

The goal of this present work was to identify the prevalence of loneliness and social isolation amongst the oldest old. High prevalence rates were identified. Heterogeneity was observed amongst the studies. The Egger tests indicated the absence of potential publication bias. Meta-regressions conducted to explore the sources of heterogeneity found that neither the assessment of loneliness nor the study continent could be attributed as significant factors contributing to the observed heterogeneity.

The prevalence of loneliness and social isolation amongst the oldest old was high compared to younger individuals. For example, Röhr found a prevalence of social isolation of 12.3% (95% CI: 11.6–13.0) based on the LSNS-6 amongst individuals aged 18 to 79 years in Leipzig Germany about ten years ago (data collection took place between Summer 2011 and Winter 2014) [50]. The prevalence of social isolation identified in this work is, for example, comparable to the prevalence amongst the frequently marginalised group of transgender individuals. This recent study identified a prevalence of 34.4% for objective social isolation based on the LSNS-6 amongst transgender individuals [51]. The data collection took place between April to October 2022. The overall high prevalence rates (for both loneliness and social isolation) of the oldest old may be attributed to the wide variety of mental and somatic disorders which are linked to this very high age [52]. Moreover, individuals aged 80 years and over have a high need for long-term care [53] (e.g. due to functional impairment). A high need for care is associated with higher loneliness levels [54]. Such individuals with a high care need may face difficulties coping with everyday life. For example, mobility restrictions could make it difficult to stay in contact with other people.

With regard to the meta-regressions, it was surprising for us that the loneliness prevalence neither varied by tool used nor the continent in which the study was conducted. This may suggest that loneliness is a general phenomenon amongst the oldest old, and may not be limited to areas or regions where, for example, individuals aged 80 years and over do not live directly or in the immediate vicinity of relatives, and where family cohesion is perhaps also differently pronounced.

Given a sufficient number of studies and data availability, future meta-analyses in this area could further explore other causes of heterogeneity. Those causes could be, for example, educational level, morbidity level or social factors (e.g. social engagement, owning a pet, grandchild care, private care receipt or spousal caregiving) [55–58]. Moreover, cultural differences, such as differences between individualistic and collectivistic societies, may be a source of heterogeneity and thus should be further explored [59].

It should be noted that the correlates of loneliness amongst the oldest old seem to be comparable to the identified correlates amongst individuals in old age [60]. For example, previous systematic reviews based on cross-sectional studies demonstrated the importance of marital status for both loneliness [60] and social isolation [61] amongst older adults.

However, great caution is required due to the overall very low number of studies investigating the correlates of loneliness amongst the oldest old. Indeed, in view of the limited number of studies examining the correlates of social isolation amongst the oldest old, it is not possible to compare it with prior findings in other age brackets (or other groups) [61].

With regards to study quality, the studies included in this meta-analysis generally exhibited a moderate to high level of methodological rigour. However, some common shortcomings were identified, such as a lack of description regarding response rates/unclear handling of low response rate. For example, a low response rate may reflect the fact that severely impaired individuals (e.g. functional or cognitive impairment) have a lower likelihood of participation; as is commonly found in cohort studies (e.g. [62]). Such impaired individuals often report higher loneliness and isolation scores compared to less impaired individuals. Thus, it may be the case that the prevalence rates reported in this work underestimates the true prevalence rates. Moreover, only very few longitudinal studies have been undertaken and even fewer have exploited the longitudinal data structure using, for example, FE estimates (which provide consistent estimates based on weak assumptions [63]). Overall, one should be very cautious about the causal interpretability of the findings, based on the available evidence.

Our systematic review and meta-analysis showed several gaps in present knowledge. There is a need for a greater number of longitudinal studies to identify the determinants of loneliness, and particularly social isolation amongst the oldest old population. In particular, we recommend the use of techniques to explore causal analysis relationships when dealing with observational data. Such techniques can include, for example, Mendelian randomisation [64, 65], matching approaches such as entropy balancing [66], cross-lagged panel models with fixed effects [67, 68], or difference-in-difference estimators [69].

Additionally, more studies based on more sophisticated tools (e.g. De Jong Gierveld tool or LSNS-6) are required. Moreover, additional studies from neglected geographic areas (particularly: Eastern Europe, South America (except for Brazil), Western Asia, South Asia, East Asia (except for China) and Africa) are required. Lastly, future studies should provide clear reporting of the response rate and should conduct a dropout analysis if necessary. Furthermore, studies are required examining the prevalence of loneliness and social isolation amongst the oldest in times of the challenging COVID-19 pandemic.

We would emphasise some strengths and shortcomings of our own work. It should be noted that this is the first systematic review/meta-analysis synthesising the prevalence and correlates of loneliness and social isolation exclusively amongst the oldest old. Additionally, important procedures were conducted by two reviewers independently. An additional hand search was performed. Furthermore, a meta-analysis and a meta-regression was conducted. A potential shortcoming is that we included solely peer-reviewed articles which may lead to the exclusion of appropriate studies. However, we decided to do so to assure a certain quality of the studies. Whilst most of the studies were published in the past few years, many more studies conducted during the COVID-19 pandemic and investigating individuals aged 80 years and over are needed. Furthermore, we restricted our search to three important databases. However, it may be the case that this choice may lead to an exclusion of studies that may be relevant. If available, other databases (e.g. Embase) should be included in the future research.

## Conclusion

Loneliness and social isolation are important problems in the oldest old. In this age bracket, further studies are required from regions outside Europe. Additionally, longitudinal studies are required to investigate the determinants of loneliness and social isolation amongst individuals aged 80 years and over. Furthermore, studies using more pronounced tools to quantify loneliness and social isolation are required.

## Supplementary Information

Below is the link to the electronic supplementary material.

Supplementary file1 (DOCX 31 KB)

Supplementary file2 (DOCX 29 KB)

Supplementary file3 (DOCX 30 KB)

Supplementary file4 (DOCX 28 KB)

Supplementary file5 (PDF 60 KB)

Supplementary file6 (DOCX 27 KB)

Supplementary file7 (DTA 27 KB)

Supplementary file8 (DO 3 KB)

## Data Availability

Please see the Additional files 7 and 8.
