# Supplementary material for: Prevalence and correlates of loneliness and social isolation in the oldest old: a systematic review, meta-analysis and meta-regression
Source: Soc Psychiatry Psychiatr Epidemiol. 2023 Dec 15;60(5):993–1015. doi: 10.1007/s00127-023-02602-0 (PMC12119783; doi:10.1007/s00127-023-02602-0)
Supplement: Supplementary file 2 — Supplementary file2 (DOCX 29 KB) [file 127_2023_2602_MOESM2_ESM.docx]

Additional File 2. Search strategy (PsycINFO and CINAHL)

| CINAHL | | |
| --- | --- | --- |
| Search ID | Search Terms | Search Options |
| S1 | “loneliness” | Expanders – Apply equivalent subjects  Search modes: Boolean/Phrase |
| S2 | “social isolation” | Expanders – Apply equivalent subjects |
| S3 | “social exclusion” | Search modes: Boolean/Phrase |
| S4 | S1 OR S2 OR S3 | Expanders – Apply equivalent subjects |
| S5 | “Oldest old” | Search modes: Boolean/Phrase |
| S6 | “Octogenarian” | Expanders – Apply equivalent subjects |
| S7 | “aged, 80 and over” | Search modes: Boolean/Phrase |
| S8 | S5 OR S6 OR S7 | Expanders – Apply equivalent subjects |
| S9 | S4 and S8 | Search modes: Boolean/Phrase |

| PsycINFO | | |
| --- | --- | --- |
| # | Searches | Type |
| 1 | exp Loneliness/ | Advanced |
| 2 | exp Social Isolation/ | Advanced |
| 3 | exp Social Exclusion/ | Advanced |
| 4 | 1 or 2 or 3 | Advanced |
| 5 | octogenarian.mp | Advanced |
| 6 | oldest old.mp | Advanced |
| 7 | (Aged, 80 and over).mp. [mp=title, abstract, heading word, table of contents, key concepts, original title, tests & measures, mesh word] | Advanced |
| 8 | 5 OR 6 OR 7 | Advanced |
| 9 | 4 and 8 | Advanced |
