# Supplementary material for: Prevalence and correlates of loneliness and social isolation in the oldest old: a systematic review, meta-analysis and meta-regression
Source: Soc Psychiatry Psychiatr Epidemiol. 2023 Dec 15;60(5):993–1015. doi: 10.1007/s00127-023-02602-0 (PMC12119783; doi:10.1007/s00127-023-02602-0)
Supplement: Supplementary file 3 — Supplementary file3 (DOCX 30 KB) [file 127_2023_2602_MOESM3_ESM.docx]

Additional File 3. Transforming loneliness measures into the categories not lonely, moderately lonely and severely lonely

| Response options in the studies | Option included in meta-analysis |
| --- | --- |
| Very lonely  Severely lonely  Always lonely  Often lonely  Most of the time lonely  Feeling lonely  Frequently lonely  UCLA-3 ≥ 6  De-Jong Gierveld (11-item version) ≥ 3 | Severely lonely |
| Sometimes lonely  Half of the time lonely  Moderately lonely  More or less lonely  Slightly lonely | Moderately lonely |
| Rarely lonely  Never lonely  Not lonely | Not lonely |

Note: Our procedure mainly follows the approach suggested by Gardiner et al. [1]

1. Gardiner C, Laud P, Heaton T, Gott M (2020) What is the prevalence of loneliness amongst older people living in residential and nursing care homes? A systematic review and meta-analysis. Age and Ageing 49 (5):748-757
