# Supplementary material for: Prevalence and correlates of loneliness and social isolation in the oldest old: a systematic review, meta-analysis and meta-regression
Source: Soc Psychiatry Psychiatr Epidemiol. 2023 Dec 15;60(5):993–1015. doi: 10.1007/s00127-023-02602-0 (PMC12119783; doi:10.1007/s00127-023-02602-0)
Supplement: Supplementary file 4 — Supplementary file4 (DOCX 28 KB) [file 127_2023_2602_MOESM4_ESM.docx]

Additional File 4. Frequency: Assessment of loneliness and social isolation

|  | Loneliness | Social isolation |
| --- | --- | --- |
| De-Jong Gierveld | 2 |  |
| Single Item | 15 |  |
| UCLA (5 items of it) | 1 |  |
| UCLA-3 | 1 |  |
| LSNS-6 |  | 3 |
| Single Item |  | 2 |
| Four activities |  | 1 |
