# Supplementary figures and images for: Prevalence and correlates of loneliness and social isolation in the oldest old: a systematic review, meta-analysis and meta-regression
Source: Soc Psychiatry Psychiatr Epidemiol. 2023 Dec 15;60(5):993–1015. doi: 10.1007/s00127-023-02602-0 (PMC12119783; doi:10.1007/s00127-023-02602-0)

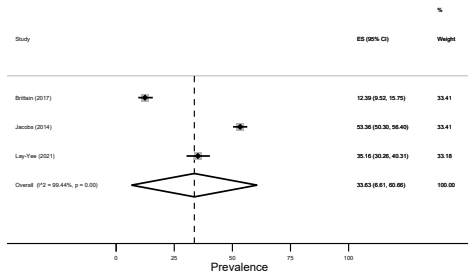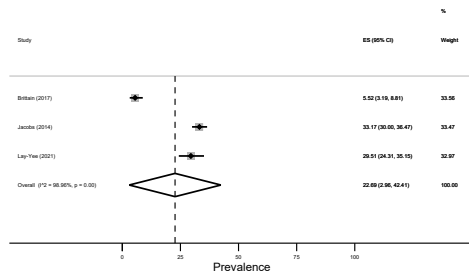

Supplement: Supplementary file 5 — Supplementary file5 (PDF 60 KB) [file 127_2023_2602_MOESM5_ESM.pdf]
