# Supplementary material for: Prevalence and correlates of loneliness and social isolation in the oldest old: a systematic review, meta-analysis and meta-regression
Source: Soc Psychiatry Psychiatr Epidemiol. 2023 Dec 15;60(5):993–1015. doi: 10.1007/s00127-023-02602-0 (PMC12119783; doi:10.1007/s00127-023-02602-0)
Supplement: Supplementary file 6 — Supplementary file6 (DOCX 27 KB) [file 127_2023_2602_MOESM6_ESM.docx]

Additional File 6. Meta-regression analysis of factors affecting heterogeneity (severe loneliness)

| Variables | Coefficient (95% confidence interval) | p value |
| --- | --- | --- |
| Assessment: - De Jong-Gierveld (Reference category: Single-item) | .76 (-.74 to 2.25) | .29 |
| - UCLA (5 items of it) | 1.43 (-1.29 to 4.14) | .28 |
| - UCLA-3 | (omitted) |  |
| Continent: - Asia (Reference category: Europe) | .04 (-1.23 to 1.32) | .94 |
| - North America | -.13 (-2.14 to 1.87) | .89 |
| - Oceania | .23 (-1.26 to 1.72) | .74 |
| - South America | .84 (-1.17 to 2.85) | .38 |

UCLA-3 was omitted because of collinearity.
